# The Impact of Filler Geometry on Polylactic Acid-Based Sustainable Polymer Composites

**DOI:** 10.3390/molecules26010149

**Published:** 2020-12-31

**Authors:** Karol Leluk, Stanisław Frąckowiak, Joanna Ludwiczak, Tomasz Rydzkowski, Vijay Kumar Thakur

**Affiliations:** 1 Faculty of Environmental Engineering, Wrocław University of Science and Technology, Wybrzeże Wyspiańskiego 27, 50-370 Wrocław, Poland; karol.leluk@pwr.edu.pl (K.L.); stanislaw.frackowiak@pwr.edu.pl (S.F.); joanna.ludwiczak@pwr.edu.pl (J.L.); 2 Department of Mechanical Engineering, Koszalin University of Technology, Raclawicka 15-17, 75-620 Koszalin, Poland; tomasz.rydzkowski@tu.koszalin.pl; 3 Biorefining and Advanced Materials Research Center, Scotland’s Rural College (SRUC), Kings Buildings, Edinburgh EH9 3JG, UK; 4 Department of Mechanical Engineering, School of Engineering, Shiv Nadar University, Uttar Pradesh 201314, India

**Keywords:** polylactic acid composites, calcium carbonate, montmorillonite, cellulose fibres

## Abstract

Recently, biocomposites have emerged as materials of great interest to the scientists and industry around the globe. Among various polymers, polylactic acid (PLA) is a popular matrix material with high potential for advanced applications. Various particulate materials and nanoparticles have been used as the filler in PLA based matrix. One of the extensively studied filler is cellulose. However, cellulose fibres, due to their hydrophilic nature, are difficult to blend with a hydrophobic polymer matrix. This leads to agglomeration and creates voids, reducing the mechanical strength of the resulting composite. Moreover, the role of the various forms of pure cellulose and its particle shape factors has not been analyzed in most of the current literature. Therefore, in this work, materials of various shapes and shape factors were selected as fillers for the production of polymer composites using Polylactic acid as a matrix to fill this knowledge gap. In particular, pure cellulose fibres (three types with different elongation coefficient) and two mineral nanocomponents: precipitated calcium carbonate and montmorillonite were used. The composites were prepared by a melt blending process using two different levels of fillers: 5% and 30%. Then, the analysis of their thermomechanical and physico-chemical properties was carried out. The obtained results were presented graphically and discussed in terms of their shape and degree of filling.

## 1. Introduction

In the plastics industry, the number of applications in which petroleum-derived plastics are replaced with biodegradable materials, also known as “green plastics”, has been rapidly growing. They are very often used in the form of biocomposites, where both the matrix and the filler are of natural origin [1,2,3,4,5]. This is related not only to the increasingly restrictive legal requirements but also to the expectations regarding the reduction of pollution of the natural environment with waste, mainly plastic waste. The global consumption of biodegradable plastics and composites is a growing market in which European countries are at the fore. The overall European consumption of such materials is well above 50% of the total world consumption. Due to the way they are manufactured, environmentally friendly materials can be divided into several groups. These include products derived directly from processed biomass (e.g., polysaccharides and proteins) and polymers produced by microorganisms (e.g., polyhydroxybutyrate, polyhydroxy valerate or bacterial cellulose). Further groups are polymers obtained by chemical synthesis: one from renewable sources (polylactic acid—PLA) and the other from various chemicals (e.g., polyvinyl alcohol, polyglycolic acid or polycaprolactone) [6,7,8]. Biopolymers are materials widely and extensively studied by scientists around the world for numerous applications, including as a potential polymer matrix for durable and biodegradable polymer composites [9,10,11,12]. One of the most widely used biodegradable polymers, which can also be described as environmentally sustainable, is polylactic acid [13,14,15]. The use of materials made of biodegradable plastics and polymer composites is currently very wide. Such materials have many applications, ranging from packaging materials (mainly for the food industry), lightweight construction materials, a wide range of materials used in medicine (e.g., implants and bioresorbable surgical threads), to parts intended for the automotive industry [16,17].

The material that is most widely used, completely biodegradable, and at the same time the most frequently researched by scientists is polylactic acid, also known as polylactide. Polylactic acid (PLA) is usually produced by fermentation from renewable agricultural materials (raw materials and waste containing starch and carbohydrates). The lactic acid molecules are formed because of lactic acid fermentation. Synthesized through this mechanism, lactic acid is a mixture of l- and d-stereoisomers, which can then be combined into a long-chain structure—PLA. One of the method of synthesizing Polylactic acid is the ring-opening polymerization (ROP) reaction. Lactic acid is first oligomerized in a polycondensation reaction and then a cyclic dimer is formed in the dehydration reaction, which is polymerized in the ROP reaction. Another method of producing PLA is the polycondensation reaction. However, the polymer obtained because of this reaction is characterized by a lower molecular weight than that obtained by ROP. The molecules formed because of polycondensation have a molecular weight of about 16,000, while ROP polymerization allows obtaining a molecular weight from 20,000 to 680,000. PLA is a linear polyester thermoplastic that can be processed similarly and in the same equipment as polyolefin and other thermoplastics. Unfortunately, it has less thermal stability as compared to other commodity polymers [7,17,18].

PLA has the softening temperature lower than the classically used thermoplastics. This is one of the limiting factors narrowing the number of its possible applications. PLA can be obtained in crystalline and amorphous forms. There are differences in the physical, thermomechanical properties, and biodegradability of crystalline and amorphous PLA. They vary depending on the structural, morphological and crystallinity changes of the polymer. For example, at high temperatures, crystalline PLA exhibits thermomechanical properties better than PLA in the amorphous state. Its softening point can be increased by using a nanofiller and producing a nanocomposite [19,20,21,22,23,24,25,26,27]. Natural fibre reinforced composites are the focus of scientists, mainly due to the ability of natural fibres to replace synthetic materials (such as glass, carbon or aramid fibres), low cost and greater affinity for the polar polymer matrix. Moreover, the low density plays a key role when using natural reinforcements instead of artificial ones. Additionally, when comparing the level of energy consumption during the production process, the production of natural fibres is more preferable [10,15,20,24,28,29,30,31,32,33,34,35,36,37,38,39,40,41,42,43,44,45,46,47,48,49,50,51].

Among the many different natural fibres, hemp and flax have been widely used for several such applications. Much attention has also been paid to the study of other natural fibres as fillers for biopolymer composites. As natural fibres are renewable, abundant and often waste, it is possible to design a fully biodegradable composite with promising properties. It can be obtained at low production cost, along with low density, appropriate fibre shape factor, high strength and specific stiffness [31,52,53,54]. Indeed, cellulose fibres are a very promising filler and are characterized by very good mechanical properties (high Young’s modulus, very good biodegradability, flexibility during processing and no abrasive properties during processing in machines), which makes them a very attractive reinforcement for the production of advanced composites [32,33]. Of course, there are also some limitations to the use of cellulose fibres in thermoplastic composites, such as poor dispersion, low thermal stability during processing, and limited compatibility with the coupling agents. [34]. Despite the above-mentioned limitations, cellulose-based nanocomposites are promising materials for numerous applications, including packaging. It is possible to increase the barrier properties while maintaining the transparency of the material.

The conducted literature studies indicate that there is a lot of data from experiments carried out on PLA composites with various fillers (including nanoclays not mentioned above), but they focus mainly on thermomechanical properties [35,36,37,38,39]. There is a lack of research and analysis concerning the influence of the filler shape on the obtained mechanical properties [55,56,57]. Therefore, in this study, the complex thermo-mechanical and physico-chemical properties obtained during the tests of composites with a polylactic acid matrix with fillers of various shapes were investigated.

## 2. Experimental

### 2.1. Materials

Investigated Polylactic acid (PLA) was acquired from NatureWorks^®^LLC. The 3051D PLA is a semicrystalline polymer, consisting of two stereoisomers: L-PLA and D-PLA, designed for injection moulding applications. According to manufacturer’s note, its physical properties are listed in the Table 1. The product was supplied in the granular form with a mean pellet dimension of approximately 3 mm.

Three types of fillers having different shapes were used in the current work. In particular, the fillers were of a different aspect ratio (length/thickness) value. These includes from the fibres (rods—the highest aspect ratio) through the hexagonal form (precipitated CaCO_3_) to montmorillonite plates. Pure cellulose fibres (purity more than 99%) were supplied by Creafill industry. Fibres of three different lengths were chosen for further investigation: 60 μm (CreaTech TC90), 700 μm (CreaTech TC700) and 900 μm (CreaTech TC900). The fibres were chosen as a filler with macroscopic-scale dimensions. The single fibre cross-section is a flat ribbon-like with dimensions (h × w): 5 μm × 20 μm. As a rectangular-like shaped nanoscale grade filler, precipitated calcium carbonate was chosen (NPCC-201). NanoMaterials Technology Pte Ltd., Singapore, supplied the filler. The average particle size was ca. 40 nm. The second nanofiller used was the montmorillonite intercalated with octadecylamine (I.30 E, PGN-T20-01, Lot No. 004). The amount of organic molecule (intercalating agent) was estimated according to the manufacturers note to 25–35%. Nanocor Inc., (Aberdeen, MS, USA) supplied the organically modified montmorillonite.

### 2.2. Sample Preparation

Using PLA polymer, the following composites with different filler ratio (wt.%) were prepared via melt blending (corresponding abbreviations in brackets are used in the following part of the paper). 5% 60 μm cellulose fibres (PLA-5C60), 5% 700 μm cellulose fibres (PLA-5C700), 5% 900 μm cellulose fibres (PLA-5C900), 30% 60 μm cellulose fibres (PLA-30C60), 30% 700 μm cellulose fibres (PLA-30C700), 30% 900 μm cellulose fibres (PLA-30C900) and 5% nanoclays (PLA-5MMT) and 5% precipitated calcium carbonate (PLA-5CaCO3) were used. As a reference sample, neat polylactic acid (PLA) was also investigated. Polymer matrix granulate, as well as fillers, were dried before processing in a vacuum dryer at a pressure of 0.4 bar. For all the materials used, the drying conditions were as follows: the temperature was 105 °C and time 3 h. Before the processing, the moisture content was measured. In all samples, the 0.1% level was not exceeded. Composites were prepared by mixing the designated amounts of polymer granulate and filling material using POLYLAB Q20 (Thermoscientific, Waltham, MA, USA) equipped with an internal mixer with a chamber volume of 50 cm. The processing conditions were set as follows: temperature 170 °C and rotational speed 60 rpm.

Mixing of components was completed after the torque reached equilibrium, which took approximately 6 min. The weighed amount of a filler was added in portions every 10 s, to ensure proper dispersion degree. For each composition, four runs were performed to provide a sufficient amount of material for further processing through injection moulding.

After mixing, the composite was milled and the granulate was introduced into the screw injection moulding machine (BOY 35A (Exton, PA, USA). The samples for mechanical testing had the following dimensions: 4 mm × 2 mm × 30 mm (for tensile strength testing) and 10 mm × 4 mm × 100 mm (bars for bending strength and Charpy impact tests). Besides, the neat polymer samples were produced in the same manner. The plates of each mixed material (as well as a polymer matrix) were created using a hydraulic press (LabTech LP-20B (Praksa, Muang, Samutprakarn 10280, Thailand)). The granulate was heated up to 170 °C and kept in an isothermal condition for 3 min. After this time, external pressure was applied for two minutes (50 bar). Then, the plate was cooled down to an ambient temperature. All plates had the following dimensions: 100 mm × 100 mm and were 1 mm thick (for mechanical testing) and 0.2 mm (for gas permeability tests).

### 2.3. Methods

Thermal analysis: TA instruments Q20 Dynamic Scanning Calorimeter was employed for thermal properties characterization (New Castle, DE, USA). Sample amount varied around 10 mg. All of the samples were investigated in the temperature range +40–+200 °C. Heating, as well as cooling rate was set to 10 °C/min. As a reference material, an empty crucible was used. Glass transition temperature, melting point and crystallinity factor were calculated from the DSC investigation. All measurements were performed under a nitrogen atmosphere.

Thermomechanical tests: The dynamic mechanical analysis was performed using TA Instruments DMA 2980 apparatus (New Castle, DE, USA). Measurements were performed in temperature range +30–+140 °C with heating rate 10 °C/min at a single frequency of 1 Hz (single cantilever mode).

Mechanical tests: All tensile and bending characteristics were carried out using Lloyd K10 tensile tester according to ISO 527 standard testing method (Worthing, West Sussex, UK)). Clamp pulling speed was 10 mm/min and the stress-strain dependency was recorded until the sample break. Strain and bend tests were repeated at least six times for each of the investigated samples.

Resilience: The resilience of the samples was investigated using falling dart impact tester CEAST DartTester (Norwood, MA, USA) and Charpy method (Resil 5.5, CEAST) (Norwood, MA, USA). The DartTester measurement proceeds with platy samples for which dimensions were mentioned above. Tests were repeated four times for each sample (DartTester) and five times for Charpy measurements.

Morphology: Morphology of the composites was characterized using scanning electron microscopy (VEGA3 LM, TESCAN) (Brno, Czech Republic). The impact test specimens were used for the analysis. Sputtering with gold (Sputter Coater Cressington 108) (Watford, UK) was performed before SEM observations. An accelerating voltage of 5 kV was used. Program VegaTC was employed for image analysis.

Thermal conductivity: coefficient was measured using THASYS equipment from Hukseflux Thermal Sensor manufacturer (Delft, Netherlands). The samples measured were plate formed with dimensions approx. 5 cm × 10 cm. The measurement temperature was 20 °C, while the measuring time was set to 900 s.

## 3. Results and Discussion

**Figure 1:**
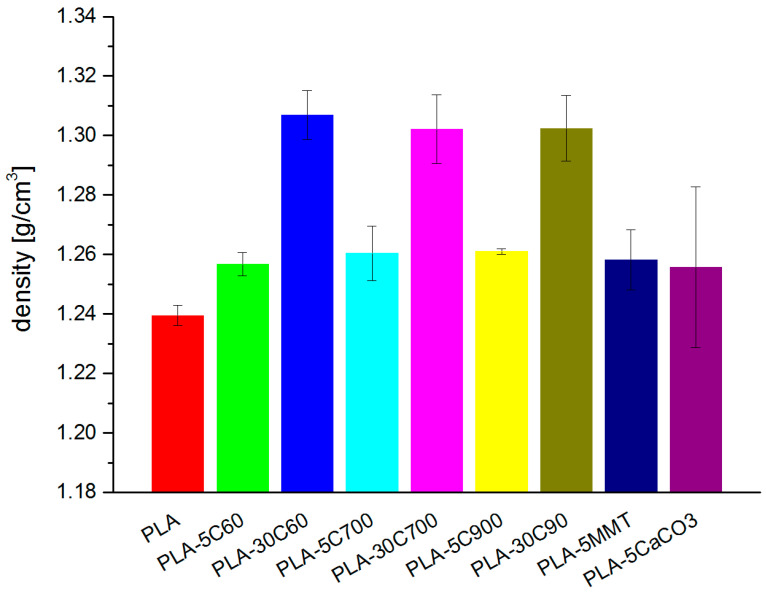
Density measured in the investigated samples.

Density is one of the most imperative properties and herein we discuss the results from density measurements. The density, measured for all the samples follows the rule of mixtures and strongly depends on filler concentration (see Figure 1).

The density for cellulose reinforced composite is increased by about 1.5% in 5 wt.% loaded composite and of 5% in composite with 30 wt.% loadings. In the group of fibre reinforced composites, observed differences among the samples with different fibres length (but with the same loading degree) are irrelevant and can be neglected.

The density of the composite samples containing 5% filler revealed the fact that loaded samples behave regardless to the filler type and its geometry (rods—fibres, plates—montmorillonite, cuboids—calcium carbonate). One of the reason may be the filler content, which occurred to be not sufficiently high to have a significant effect on the measured physical parameter. Moreover, the densities of all three fillers (cellulose, montmorillonite, calcium carbonate) are similar, so their impact on the composite density is rather minimal.

Using the value of density for each of the component (polymer matrix and a filler), as well as the established percentage contribution in the composite, a theoretical composite’s density was calculated. The calculations were performed based on the rule of mixtures assumptions. Table 2 represents resulting values—in the last column. There is a percentage difference between two values (calculated concerning measured value).

The observed discrepancies between a theoretical and measured value of a composite density are less than 1% (for low-loaded composites) and reach almost 6% for highly loaded cellulose reinforced composites (irrespective to the fibre length). Focusing on the system with the highest result inconsistency (PLA-30C), it may be concluded that such a system does not fully follow the simple rule of mixtures. Nevertheless, there may be an additional explanation. The formation of free volume voids in the processed composite may occur in the bulk material as well as at the fibre/polymer interface. The higher the filling degree, the more voids can be formed because of poor mixing efficiency (and thus more anisotropic filler distribution) thus influencing material’s properties.

**Figure 2:**
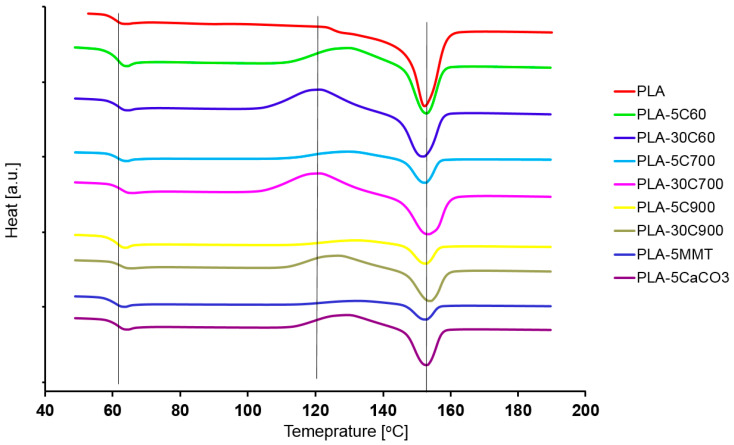
Dynamic Scanning Calorimetry measurements for all of the investigated samples.

**Figure 3:**
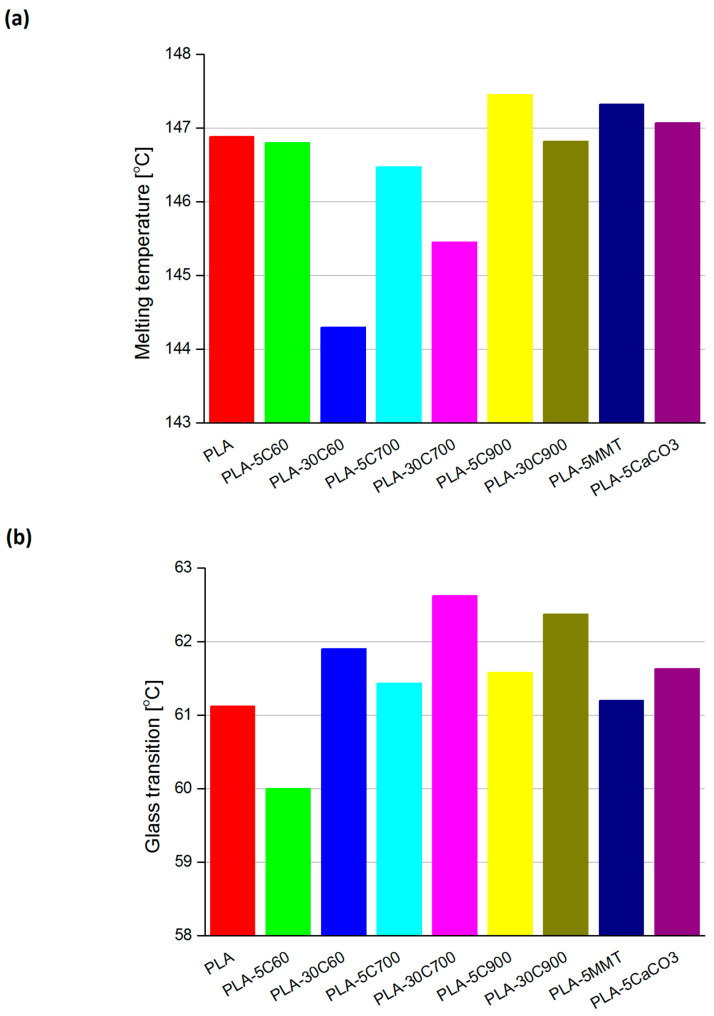

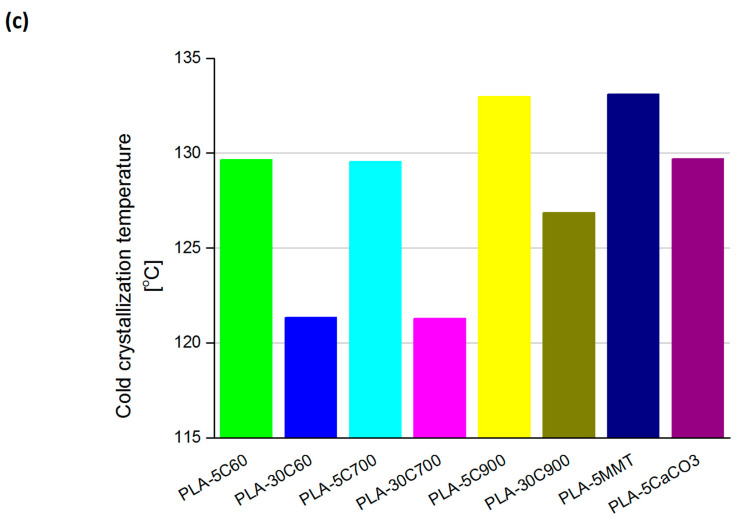
Melting temperatures (**a**) glass transition (**b**) and cold crystallization (**c**) for all of the investigated composites. Cold crystallization temperature was not recorded for PLA sample.

**Figure 4:**
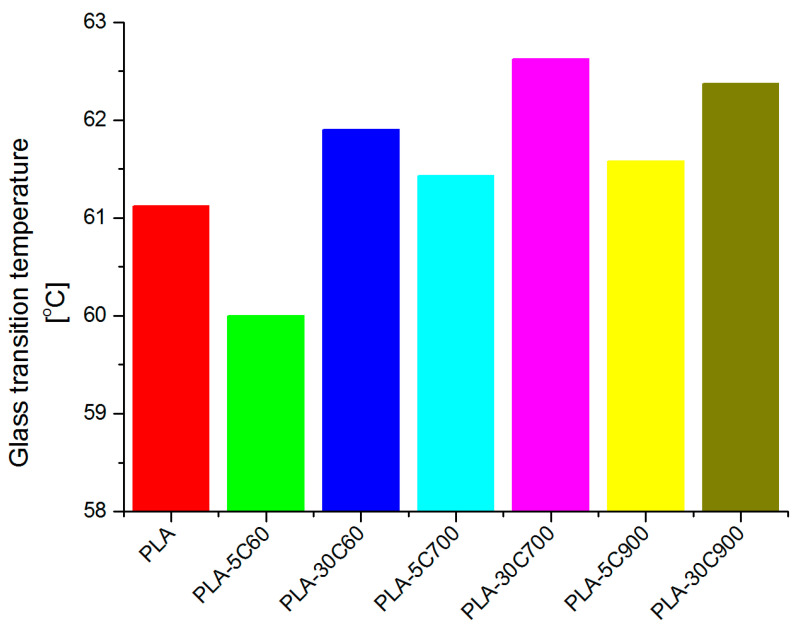
Comparison of glass transition temperatures obtained for low and highly loaded fibre reinforced PLA composites.

Figure 2 presents the DSC runs obtained for all of the samples. Three straight, vertical lines indicate three regions (on the temperature axis) where characteristic temperatures are expected to exist. Starting from the low-temperature region, these are in particular: glass transition, cold crystallization and melting temperature. With respect to the pristine polymer resin, the regions were chosen arbitrarily. Figure 3 compares the indicated temperatures that were extracted. Two plots on Figure 4 compare the glass transition temperatures selectively for low- (a—left) and highly loaded (b—right) fibre reinforced composites.

Glass transition as well as cold crystallization temperatures were measured in the corresponding inflexion point, whereas melting temperature was analyzed from the peak onset. The signal related to cold transition process was not always easy to determine, as for some samples (like PLA, PLA-5C700, PLA-5MMT) the corresponding run was almost flat in the discussed region (see Figure 2).

Focusing the attention on the analysis of the glass transition (Figure 3b) for cellulose fibre composites, a logical observation can be withdrawn. Introducing fibres with a larger aspect ratio (increasing their longitude dimension) leads to a slight shift in the transition temperature. The situation is highlighted for both the 5% and 30% filled composites (compare additional plots on Figure 4). What is interesting, in the case of PLA-5C60 sample, the glass transition temperature is lower than in pristine polymer. With PLA-5C700 and PLA-5C900 samples, the temperature rise was estimated to (respectively) 0.5 and 0.8%. For highly-loaded cellulose composites, the increase was as follows: 1.3% (PLA-30C60), 2.5% (PLA-30C700), 2% (PLA-30C900). For montmorillonite and calcium carbonate filled composites, results are similar to cellulose low-loaded composites and are respectively: 0.1% and 0.8%. The dependency recorded for cold crystallization temperature has much in common with the results discussed above. Looking at the corresponding plot in Figure 3, one can notice that the measured temperature is slightly increasing with the fibre filler aspect ratio. In the samples belonging to a series of different filling degrees (5% and 30%), the first two results are comparable, only the third one is significantly higher. As for unfilled PLA, there was no measurable signal referring to cold crystallization temperature, the composite sample with the shortest fibres is assumed as a reference one. So that the relative increase reached the level of 2.6% and 4.5% (for PLA-5C900 and PLA-30C900 samples respectively).

The cold crystallization temperatures in PLA-5MMT and PLA-5CaCO3 composites indicate similarities to those recorded for high loaded fibre composites. The last parameter measured in the DSC experiment was melting temperature. This parameter seems to be rather stable in all investigated samples, excluding PLA-30C60 and PLA-30C700 samples. The reason may be the unsymmetrical character of the signal. For these two samples, the peak is influenced by the nearby lying signal corresponding to the sample’s crystallization process (see Figure 2). So that the onset point would be shifted to the lower temperatures as, it can be observed on the discussed plot.

**Figure 5:**
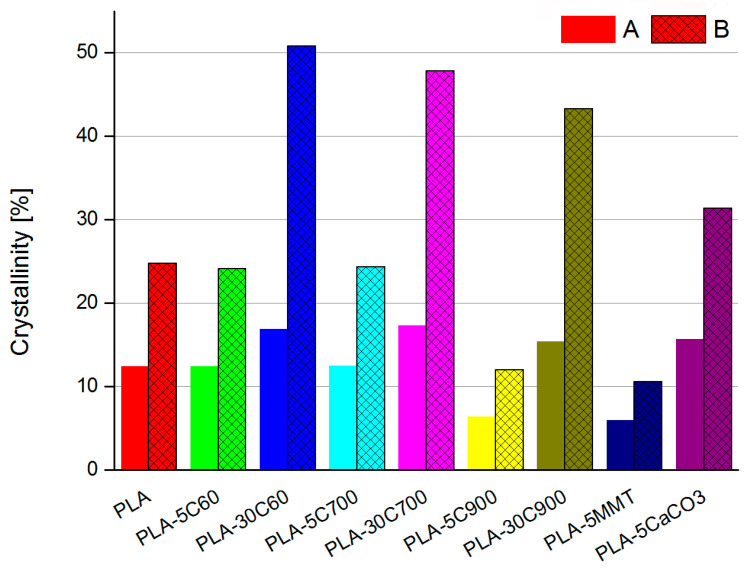
Comparison of the crystallinity calculated using two different ways. A—heat of fusion, crystallization as well polymer to filler ratio taken into consideration; B—simple calculation relying only on heat of crystallisation

Following Liu et al. [40] PLA crystallinity factor was calculated in all of the investigated samples (“A” series on Figure 5). For such calculation, enthalpies of fusion and crystallization, as well as polymer matrix weight ratio had to be known. The result was then compared with the simple calculations (available from the DSC apparatus menu) considering only the heat of crystallization of the investigated material (“B” series on Figure 5). Comparing the crystallinity calculated using two different ways, the values obtained for the model introduced in the work of Liu et al. [40] are approximately 2 times higher, comparing to the simple calculation. Nevertheless, what is important, in the qualitative way they are comparable, irrespective of the calculation method.

Introducing a low amount of fibre filler does not change the composite’s crystallinity. Even, taking into consideration the fact of fibre—induced crystallization, it seems to be an obvious and non-surprising observation. The interesting thing is a strongly marked increase of the crystallinity for a highly loaded sample. The direct reason is the high value of cold fusion in the respective samples, which may be a result of an increase in the number of nucleation regions. Introducing a considerable amount of a filler into the polymer matrix may have two opposite effects. From one side, it is expected to decrease the crystallinity, but due to the increase of nucleation regions (located on the fibre/matrix interface), the crystallinity can even increase. These opposing phenomena were discussed in the author’s earlier work, in terms of flax reinforced polycaprolactone composites [41]. The crystallization process may occur in the polymer matrix-cellulose fibre interface thus increasing the crystallization rate and as a result the value of the sample’s crystallinity index. In the non-rod filled composites, the crystallinity parameter is decreased by a factor of 2.5 for the clay composite sample but increased (ca. 20%) for the calcium carbonate filled composite, comparing to the polymer matrix sample (compare—Figure 5).

In the composites filled with non-rod like fillers, the observed changes in the characteristic temperatures (like cold crystallization, melting and glass transition), if any, are minor (see Figure 2 and Figure 3). A much more interesting change in the crystallinity index—for composite filled with platy one, the parameter has decreased 2.5 times whereas for cuboid material the parameter has increased by 20% compared to pristine polymer sample. The effect of nucleation and an increase in the degree of crystallinity after adding CaCO_3_ [58] and other fillers are known and described in the literature [59]. The difference is due to the aspect ratio of montmorillonite plates, which are in general much thinner than calcium carbonate filler. Therefore, the montmorillonite plates can build up within the polymer matrix structure in various ways. That may be simple embedding the filler (a stack of the aluminosilicate layers), polymer intercalation (single polymer chains are incorporated in-between the two adjacent silicate layers) and exfoliation of the filler platelet. The last stage is desirable in terms of creating the non-permeable barrier within the polymer matrix.

**Figure 6:**
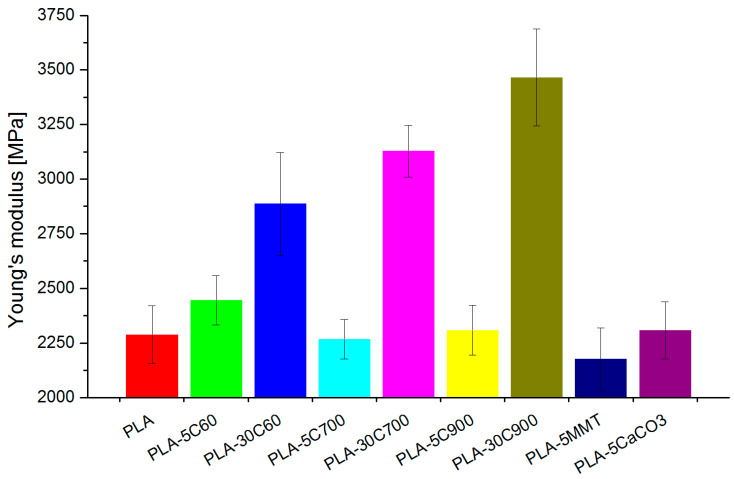
Young modulus—tensile measurements.

**Figure 7:**
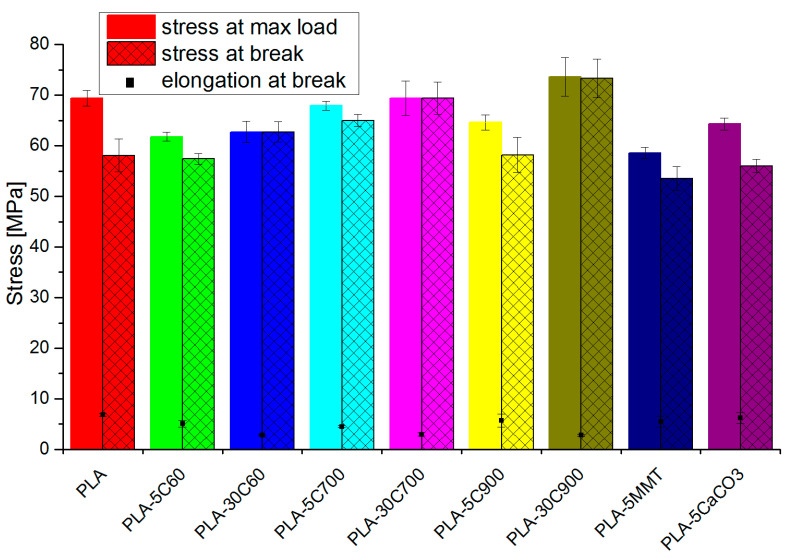
Tensile strength and stress (bars) and elongation at break (rectangular points).

Introducing the fibres into the composite increases Young’s modulus (tensile and bending test) respectively to the filling degree and fibre length (see Figure 6). The reinforcing aspect is pronounced in highly loaded composites. In addition, some dependency between the fibre longitude and Young’s modulus value can be withdrawn, but (especially for 5 wt.% composites) is of marginal importance. In comparison to the pristine polymer, in the fibre-reinforced composites, stress at break has increased, almost reaching the value for tensile strength. As the difference between tensile strength and stress at break parameters has been reduced (in 30 wt.% loaded composites—almost to zero), the elongation at break value has also followed the dependency (for composites of the brittle character reaching the lowest value)—see Figure 7.

**Figure 8:**
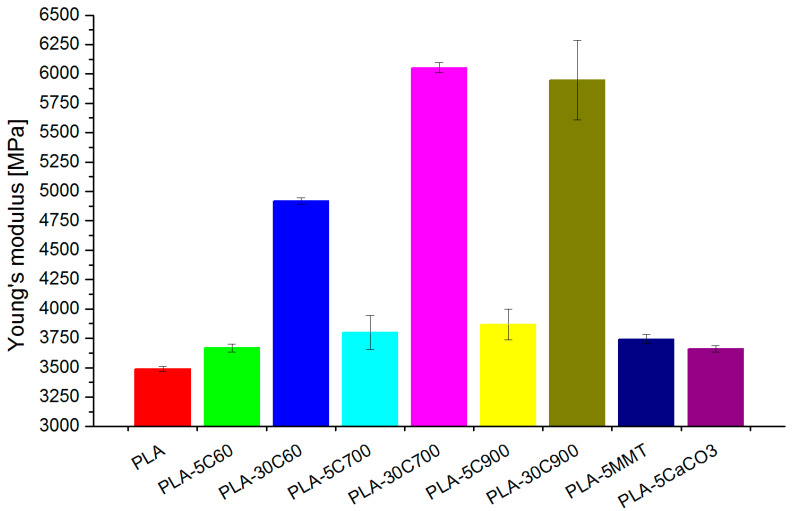
Young modulus—bending mode measurements.

Similarly, at bending measurements, Young’s modulus increased with the fibre length. The increment rate for 5 wt.% fibre composites is observable, but not that contrary to composites with 30 wt.% loadings (Figure 8).

**Figure 9:**
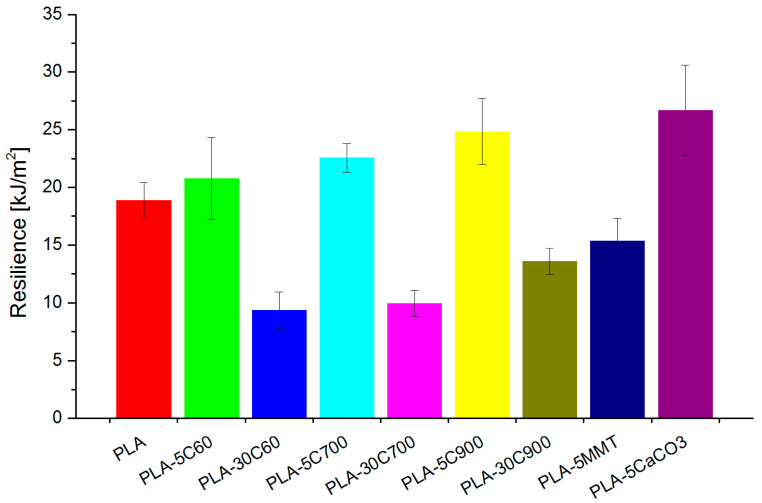
Charpy impact measured for all investigated samples.

An interesting observation can be acquired from resilience measurements (Charpy). In low loaded fibre reinforced composites, the introduction of cellulose fibres increases the parameter with the fibre length. For highly loaded composites, the resilience is two times lower (comparing to pristine polymer) but is also increasing consequently with the fibre length and is presented in Figure 9. It is a known phenomenon that in a randomly dispersed fibre reinforced composites, and under impact force of pendulum, the crack propagation can be reduced. Similar behaviour can be implemented to PLA/flax composites where the fibre surface prevents further crack propagation in the composite structure. This is true for low filler loadings as for higher loadings a noticeable decrease in resilience can be explained by crack propagation not reaching a single fibre but rather the fibre bundles present in the polymer matrix, therefore, reducing the overall impact strength of the material. Destroying fibre bundle by splitting into smaller ones or even single fibres/microfibrils can be beneficial as it would absorb part of the energy however if a bundle is pulled out during the breakage no such strength improving behaviour can be observed.

Composites containing NPCC have presented the highest resilience values and based on the literature data it can be described by nanoparticles acting as stress concentrators and therefore increasing the impact strength of polymer matrix through the crazing mechanism [60]. Where due to the specific filler shape the process of craze growth precedes cracking and allows to absorb fracture energy and therefore effectively increasing the fracture toughness.

**Figure 10:**
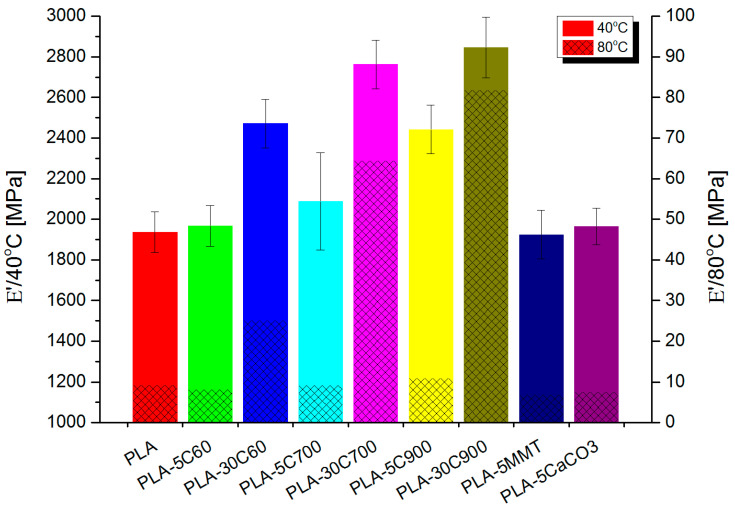
Storage moduli in obtained for all investigated samples.

DMTA investigation (Figure 10) has revealed similar dependencies (in loss and storage modulus) like in tensile measurements. For fibre-reinforced composites, the introduction of subsequent portions of cellulose fibres increases gradually the storage moduli in both glassy and rubbery state. The only exception is E’ value for highly loaded composites reinforced with 700 μm fibres.

To estimate the effectiveness of a filler incorporated into the polycaprolactone matrix on the *E*’ modulus, “*C*” coefficient was calculated [28,42] according to the formula presented below [43,44]:

(1) $$C=\frac{E_{g}^{\prime comp}/E_{r}^{\prime comp}}{E_{g}^{\prime PLA}/E_{r}^{\prime PLA}}$$

where “*E*” is storage modulus measured in glassy/rubbery state (respectively g/r lowerindex) for composite (*comp*—upper index) or polymer matrix (*PLA*—upper index).

**Figure 11:**
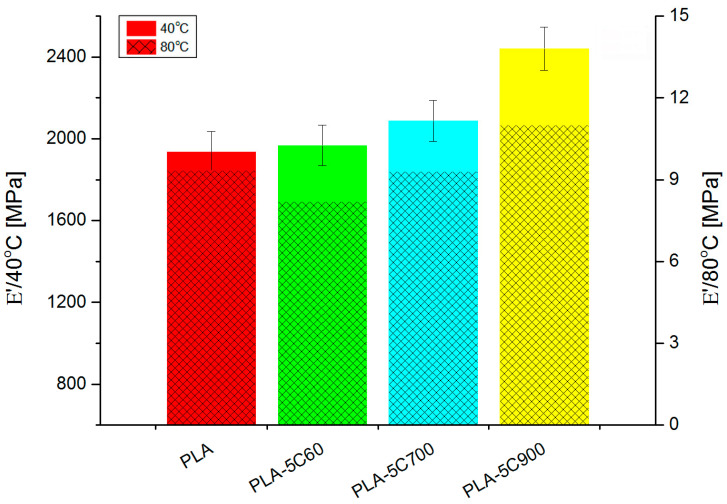
Storage modulus for cellulose fibre reinforced composites—low loading.

**Figure 12:**
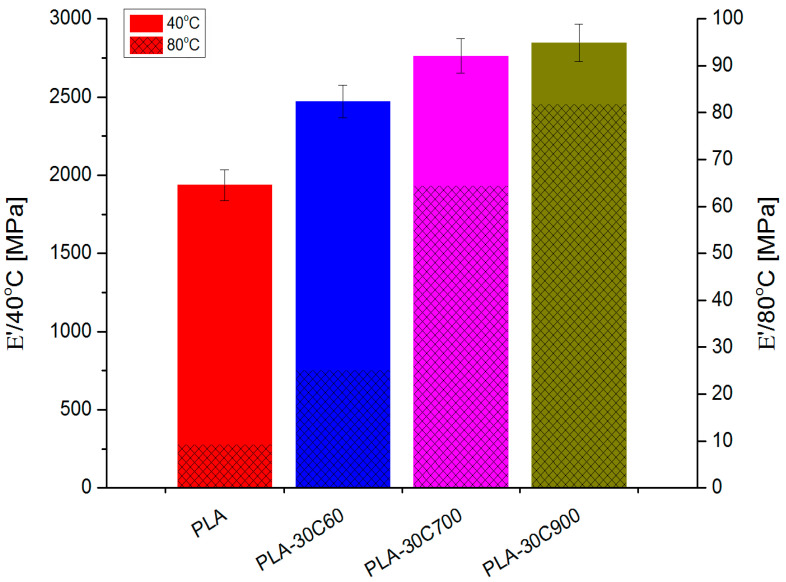
Storage modulus for highly—loaded cellulose fibre reinforced composites.

Comparing to the composite with 60 μm fibres, the storage modulus (in the rubbery state) has increased 2.5 times being the only “jump” in the discussed series of samples. Figure 11 and Figure 12 depicts this situation in a more detailed way.

For all the samples, a “*C*” coefficient (reflecting the fibre reinforcement efficiency) was calculated following the method proposed by authors in the following literature [28,42]. Table 3 presents the calculated data.

The lowest values of the coefficient (indicating the most effective behavior) was observed for two composites (30 wt.% loadings with the longest fibres: 700 μm and 900 μm). The composite reinforced with the shortest fibres reveals a higher value of the coefficient, but it has to be stated that, compared to other samples—it is still quite low.

**Figure 13:**
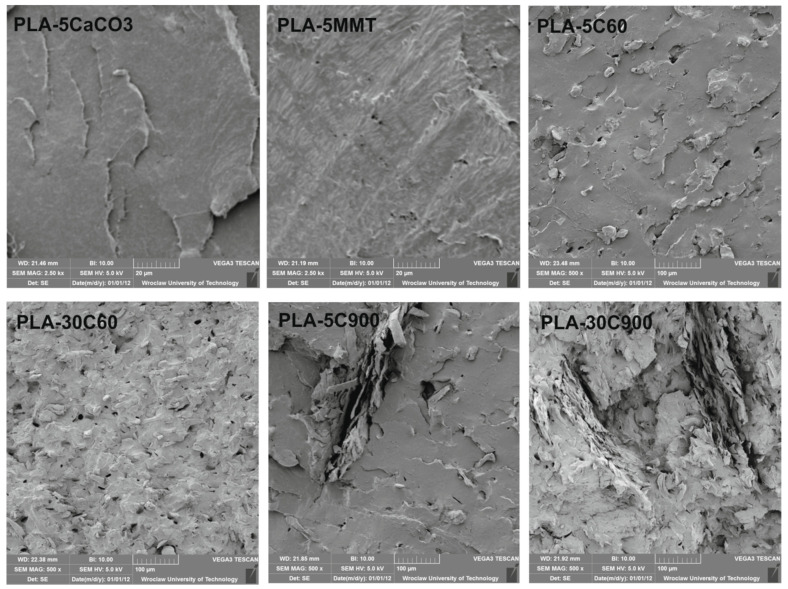
SEM images of PLA composites.

Representative SEM images of tested materials are shown in Figure 13. Due to the different dimensions of the fillers, various magnification was used, 2.5 k× and 500× respectively. Figure 13 shows filler particles of different sizes embedded in the polymer matrix as well as holes left by the particles that detached from the PLA matrix during fracture. Figure 13 illustrates the dispersion of fillers particles in composites. Particles below micro-scale (40–50 nm) can be observed in PLA composites filled with CaCO_3_, while MMT plates are about a few microns long. The cellulose has a size of 50–60 microns (C60) and larger cellulose filler particles (700–900 µm) in the case of C900 are visible on the surfaces of the materials. It turns out that, for cellulose, MMT, and CaCO_3_ fillers, good dispersion in the PLA matrix was obtained.

**Figure 14:**
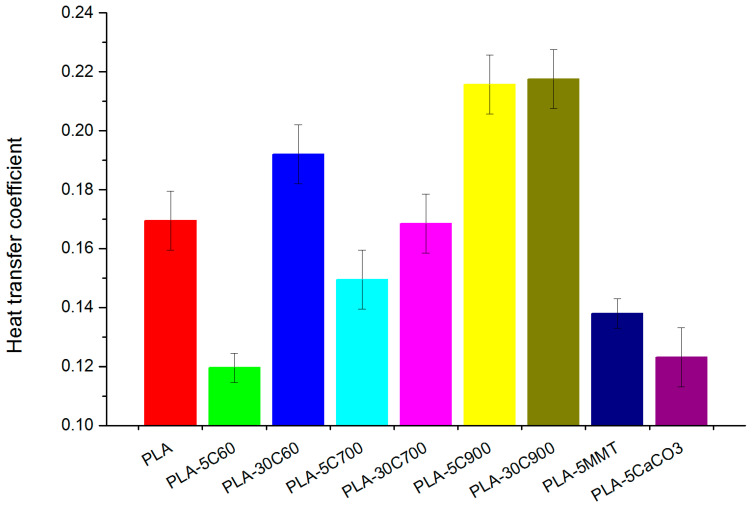
Heat transfer coefficient in the PLA and its composites.

Heat transfer through composite is preferred in fibre reinforced samples, what might be due to fibre orientation producing a composite with pronounced isotropic character—Figure 14. The observed increase in the heat transfer coefficient for the high concentration of cellulose fibres can be explained by water uptake which is a known phenomenon and was reported in the literature [61]. In contrast, cubic and platy fillers, as well as short fibres sample, revealed the lowest coefficient—30% below the value for the pristine polymer. The fibre alignment is consistent with tensile results (compare Figure 10).

**Figure 15:**
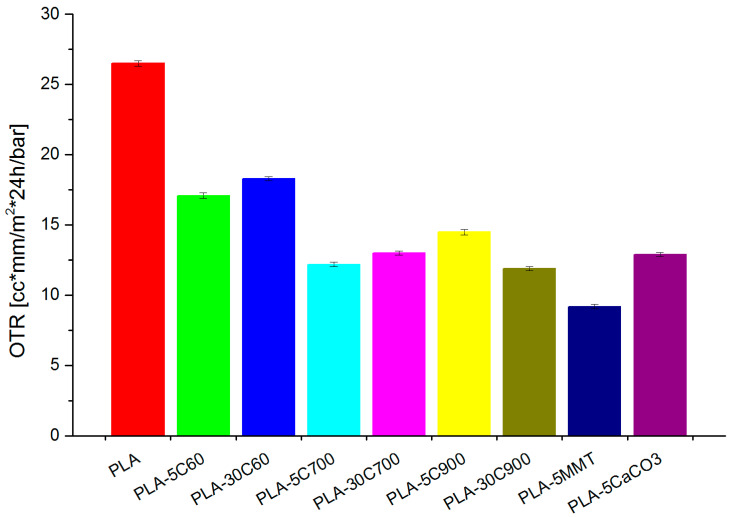
Oxygen permeability measured for investigated samples.

As the last test, oxygen permeability results have also been discussed (Figure 15). Oxygen permeability measurements revealed that all fillers dampened oxygen transfer through polymer film. As it was supposed, montmorillonite increased barrier properties most effectively. The results are as expected as the microscopic images (Figure 13) showed that the MMT plates were well dispersed in the matrix which resulted in a lower gas permeability through the material. On the other hand, the shortest cellulose fibres have the weakest influence on the measured property. Apart from these facts, there is no significant difference among fillers influence on permeability.

## 4. Conclusions

1. Different filler types influence on polylactide acid matrix has been presented. The results obtained in this study showed that the aspect ratio of an introduced filler plays a key role in the material end properties.
2. Mechanical properties are highly affected by the filler loading and fibre length.
3. Enhanced barrier properties, attractive for packaging purposes, were received due to dispersion of montmorillonite nanoparticles in PLA.
4. Fibrous composites (long fibres) reveal signs of filler alignment that was pronounced in thermal and tensile measurements.

## Figures and Tables

**Table 1:** Some of the PLA physical properties (following manufacturer’s note).

Parameter	Value	Unit
Melting temperature	150–165	°C
Glass transition temperature	55–65	°C
MFR (2.16 kg, 210 °C, std die)	10–25	g/10 min
Clarity	Transparent	-

**Table 2:** Calculated (relating to the rule of the mixture) and measured densities.

Sample	Theoretical	Measured	Δ (%)
PLA-5 °C	1.262	1.26	0.4
PLA-30 °C	1.384	1.31	5.9
PLA-5MMT	1.264	1.26	0.5
PLA-5CaCO3	1.245	1.26	0.9

**Table 3:** Storage moduli and fibre reinforcement efficiency for all samples.

E’	40 °C	80 °C	C Coeff
PLA	1936	9.3	-
PLA-5C60	1968	8.2	1.2
PLA-30C60	2472	25	0.5
PLA-5C700	2088	9.3	1.1
PLA-30C700	2763	64	0.2
PLA-5C900	2442	11	1.1
PLA-30C900	2846	82	0.2
PLA-5MMT	1924	6.9	1.3
PLA-5CaCO3	1965	7.6	1.2

## Data Availability

Data contained within the article are available on request from the authors.

## Sample Availability

Samples of the all presented polymer compounds are available from the authors.
